# Internet of Things-Assisted Smart Skin Cancer Detection Using Metaheuristics with Deep Learning Model

**DOI:** 10.3390/cancers15205016

**Published:** 2023-10-17

**Authors:** Marwa Obayya, Munya A. Arasi, Nabil Sharaf Almalki, Saud S. Alotaibi, Mutasim Al Sadig, Ahmed Sayed

**Affiliations:** 1 Department of Biomedical Engineering, College of Engineering, Princess Nourah Bint Abdulrahman University, P.O. Box 84428, Riyadh 11671, Saudi Arabia; 2 Department of Computer Science, College of Science and Arts in RijalAlmaa, King Khalid University, Abha 62529, Saudi Arabia; 3 Department of Special Education, College of Education, King Saud University, Riyadh 12372, Saudi Arabia; 4 Department of Information Systems, College of Computing and Information System, Umm Al-Qura University, Mecca 21421, Saudi Arabia; 5 Department of Computer Science, College of Science, Majmaah University, Al Majmaah 11952, Saudi Arabia; 6 Research Center, Future University in Egypt, New Cairo 11835, Egypt

**Keywords:** Internet of Things, skin cancer diagnosis, dermoscopic images, deep learning, metaheuristics

## Abstract

**Simple Summary:**

The Internet of Things (IoT) uses connected devices and sensors, like high-resolution cameras and specific sensors in wearable devices, for the collection of skin images with abnormalities. Skin cancer detection is difficult because of differences in lesion size, shape, and lighting conditions. To address this, an innovative approach called “ODL-SCDC”, combining deep learning with IoT technology, is developed. The proposed model uses advanced techniques like hyperparameter selection and feature extraction to improve skin cancer classification. The results show that ODL-SCDC outperforms other methods in accurately identifying skin lesions, which could have a significant impact on early cancer detection in the medical field.

**Abstract:**

Internet of Things (IoT)-assisted skin cancer recognition integrates several connected devices and sensors for supporting the primary analysis and monitoring of skin conditions. A preliminary analysis of skin cancer images is extremely difficult because of factors such as distinct sizes and shapes of lesions, differences in color illumination, and light reflections on the skin surface. In recent times, IoT-based skin cancer recognition utilizing deep learning (DL) has been used for enhancing the early analysis and monitoring of skin cancer. This article presents an optimal deep learning-based skin cancer detection and classification (ODL-SCDC) methodology in the IoT environment. The goal of the ODL-SCDC technique is to exploit metaheuristic-based hyperparameter selection approaches with a DL model for skin cancer classification. The ODL-SCDC methodology involves an arithmetic optimization algorithm (AOA) with the EfficientNet model for feature extraction. For skin cancer detection, a stacked denoising autoencoder (SDAE) classification model has been used. Lastly, the dragonfly algorithm (DFA) is utilized for the optimal hyperparameter selection of the SDAE algorithm. The simulation validation of the ODL-SCDC methodology has been tested on a benchmark ISIC skin lesion database. The extensive outcomes reported a better solution of the ODL-SCDC methodology compared with other models, with a maximum sensitivity of 97.74%, specificity of 99.71%, and accuracy of 99.55%. The proposed model can assist medical professionals, specifically dermatologists and potentially other healthcare practitioners, in the skin cancer diagnosis process.

## 1. Introduction

The Internet of Things (IoT) is designed by interconnecting devices to the Internet using modern communication technology for sharing data [1]. Recently, IoT has been popularly implemented in various appliances such as vehicular ad hoc networks, smart grids, body sensor networks, smart cities, and smart homes [2,3]. The IoT development depends on diverse advanced technologies, namely, wireless sensor networks (WSNs), cloud computing (CC), and information sensing [4]. The IoT is usually exploited to enhance and develop medical systems due to its effective power for integrating with the resources of substructures and offering essential data to users [5]. The medical system puts a considerable quantity of data through WSNs when distributing various e-health services, namely, electronic health records, remote monitoring for patients, and medical platforms [6]. Skin cancer is considered the sixth major cancer variety, which could be improved around the world. The skin layer comprises three forms of cells, melanocytes, basal cells, and squamous cells, in which cells are responsible for tissues to induce cancer [7,8]. Hence, there are different skin tumors, namely, basal cell carcinoma (BCC), melanoma, and squamous cell carcinoma (SCC), which can be a serious variety of cancers. People are mainly affected by skin cancer in Australia and the United States [9]. Diagnosis of skin cancer at an earlier phase is challenging for dermatologists, which stimulates research workers to develop a simplified and automated cancer detector for identifying skin cancer at an earlier phase [10].

Dermoscopy improves melanoma diagnostic accuracy; however, it can be quite difficult to accurately analyze some cancers, and especially earlier melanomas have insufficient special dermoscopic features [11]. Although dermoscopy analyzes skin cancers with better accuracy, it is not appropriate for identifying featureless melanoma, and it requires increased accuracy to improve the patient’s survival rates [12,13]. The difficulties with dermoscopy and the requirement to enhance the identification accuracy of skin tumors then positions the substructure for emerging computer-aided detection (CAD) techniques for analyzing skin cancers [14]. In general, there have been five stages in computer-aided skin cancer analysis such as feature extraction, segmentation, classification, preprocessing, and image acquisition [15]. The important stages in the CAD of skin cancers are classification and segmentation [16]. But, identifying skin cancer employing CAD is simple, and we should consider numerous aspects for accurate identification, for instance, artefacts like ruler signs, dark corners, ink marks, water bubbles, hairs, and marker signs, which may lead to incorrect segmentation and misclassification of skin cancers [17,18]. In several computer-aided techniques, deep learning (DL)-based algorithms provide optimistic outcomes for the classification and segmentation of skin cancers due to their capability for extracting complex features from skin cancer images for extremely specific diagnosis [19]. Also, DL methods learn function-specific features and are more effective than other techniques.

This article presents an optimal deep learning-based skin cancer detection and classification (ODL-SCDC) algorithm in the IoT environment. The goal of the ODL-SCDC technique is to exploit metaheuristic-based hyperparameter selection approaches with a DL model for skin cancer classification. To achieve this, the ODL-SCDC technique undergoes preprocessing using a Wiener filtering (WF) system. Moreover, the ODL-SCDC algorithm involves an arithmetic optimization algorithm (AOA) with an EfficientNet model for feature extraction. For skin cancer detection, a stacked denoising autoencoder (SDAE) classification model has been used. Lastly, the dragonfly algorithm (DFA) is utilized for the optimal hyperparameter selection of the SDAE algorithm. The simulation validation of the ODL-SCDC algorithm can be tested on a benchmark skin lesion database. The key contributions of the paper are summarized as follows.

- Develop an automated ODL-SCDC technique comprising WF-based preprocessing, AOA with EfficientNet-based feature extraction, SDAE classifier, and DFA-based hyperparameter tuning. To the best of our knowledge, the proposed ODL-SCDC technique never existed in the literature.
- Propose AOA with the EfficientNet model for feature extraction, a critical aspect of skin cancer classification. The AOA-based fine-tuning process is crucial for optimizing the performance of the classification model.
- Present an SDAE classifier for skin cancer classification and DFA is employed for optimal hyperparameter selection of the SDAE model. Hyperparameter optimization of the SDAE model using DFA using cross-validation helps to boost the predictive outcome of the proposed model for unseen data.

## 2. Related Works

In [20], a powerful skin cancer identification model was presented for enhancing accuracy by learning and extracting significant image representations through a MobileNetV3 framework. Subsequently, the removed features were employed as input to an adapted Hunger Games Search (HGS) based on Dynamic-Opposite Learning (DOLHGS) and PSO. Ramya and Sathiyabhama’s [21] primary aim was creating an ensemble ML with an improved genetic algorithm (GA) method for attaining high-level accurateness in the prognosis of skin cancers at an early phase by comparison with other present methods. Then, the feature selection (FS) was implemented by utilizing an Enhanced-GA (EGA) that generates enhanced solutions through processes such as ensemble, mutations, and crossover with ELM (EGA-ELM) for classifying the images as non-cancerous or cancerous. Abd Elaziz et al. [22] designed a robust technique for skin cancer diagnosis with a DL-based algorithm as the extracted features support that a diagnosis could be attained by employing the MobileNetV3 framework. Further, an innovative technique named the Improved Artificial Rabbits Optimization (IARO) was presented that exploits the crossover operator and Gaussian mutation to avoid the irrelevant features from the feature extraction by the MobileNetV3 framework.

Khamparia et al. [23] introduced a new DL Internet of Health and Things (IoHT)-determined model for classifying skin cancers in skin images by implementing the TL method. In this developed model, automated features are removed from images employing various pretrained frameworks, namely, SqueezeNet, VGG19, Inception V3, and ResNet50, that were provided in the fully connected layer (FCL) of a CNN for the classification of malignant and benign skin cells utilizing a dense and max pooling process. The authors of [24] suggested a novel skin cancer detection technique named DL with Evolutionary Algorithm Image Segmentation (DL-EAIS) for IoT and cloud-based smart medical fields. Firstly, dermoscopic images could be taken by employing IoT devices that must be transferred to cloud servers for additional identification. Secondly, the shallow CNN (SCNN) framework was exploited for feature extraction. Moreover, the Deep-Kernel-ELM (D-KELM) algorithm has been utilized as a classification technique for identifying the class labels of dermoscopic images. In [25], the DL technique (CNN) was utilized to develop a computer technique to forecast novel conditions of skin cancers. Later, this developed method made a CNN approach that contains four fully connected layers, three convolution layers, and three max pooling layers. Adjobo et al. [26] implemented a Gabor Convolutional Network (GCN) method to enhance the effectiveness of the automatic method of analysis for skin tumors. This algorithm integrates a CNN and Gabor filtering (GF) and supports three operations such as the collection of GF banks, a CNN model, and filter injection. In [27], a DL-assisted hybrid optimizer was employed to identify skin cancer and segmenting lesions. Two optimization techniques have been implemented for diagnosing cancers and segmenting skin lesions. MultiScale Residual Fusion Network (MSRFNet) was exploited for the segmentation of skin cancer and could be trained by the developed Average Subtraction Student Psychology-Based Optimizer (ASSPBO) technique.

## 3. The Proposed Model

**Figure 1:**
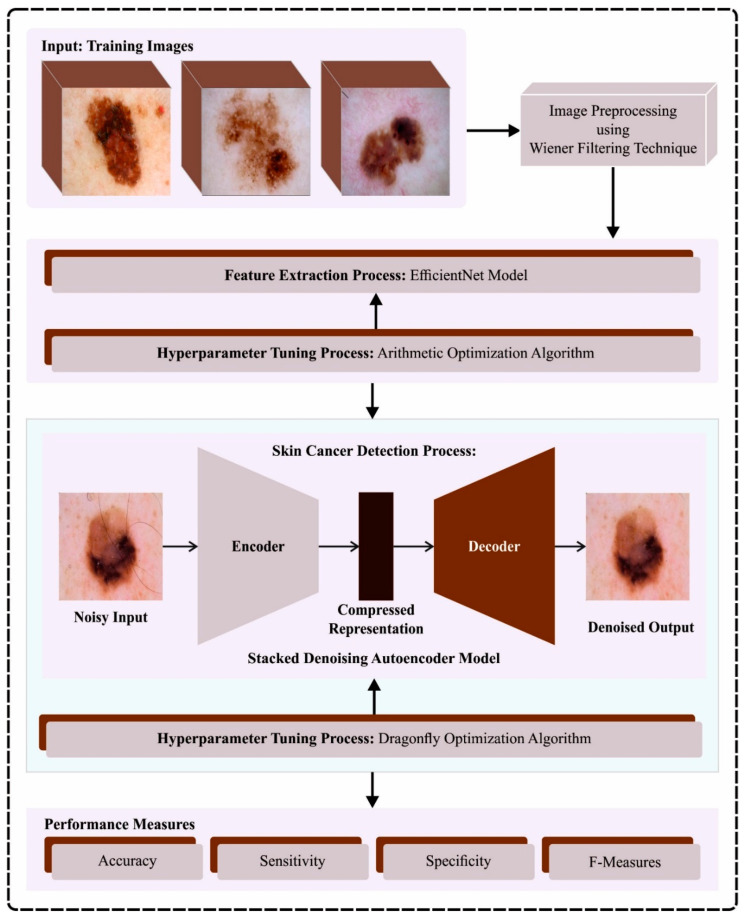
Overall process of ODL-SCDC algorithm.

In this article, we have designed and developed an automated skin cancer classification and detection model using the ODL-SCDC technique in the IoT environment. The goal of the ODL-SCDC technique is to exploit metaheuristic-based hyperparameter selection approaches with a DL model for skin cancer classification. To achieve this, the ODL-SCDC technique performs a series of processes such as WF-based processing, EfficientNet-based feature extraction, AOA-based hyperparameter tuning, SDAE-based classification, and DFA-based parameter tuning. Figure 1 depicts the entire process of the ODL-SCDC approach.

### 3.1. Image Preprocessing

To preprocess the input images, the WF approach is used. The WF is named after Norbert Wiener, and it is a mathematical model for signal processing and filtering [28]. It is mainly utilized in the domains of statistics, engineering, and image processing for estimating an unknown signal or system by decreasing the mean squared error (MSE) among the evaluated signal and true signal. The WF is extremely beneficial if dealing with noisy signals or once the features of noises are known. Mathematically, the WF can plan for minimizing the MSE among the estimation signal and true signal. It usually contains convolutional, spectral analysis, and statistical estimates. The filtering is executed in either the time or frequency domains, based on the nature of the problems and the existing data.

### 3.2. Feature Extraction Using EfficientNet Model

**Figure 2:**
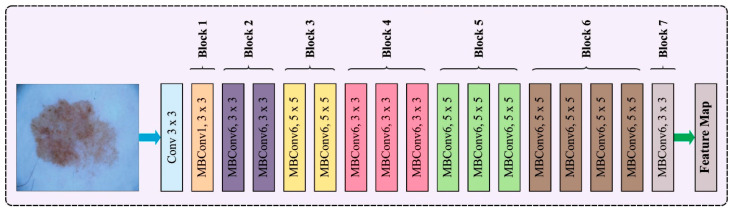
Architecture of EfficientNet.

In this work, the EfficientNet approach is applied for feature extraction. A model scaling algorithm is used to enhance the accuracy and speed of the model. To accomplish this, different sizes of scaling models can be re-examined as suggested by the predecessors, involving the width, depth, and resolution of the network [29]. The researchers recognized that the dimension is mutually influential and EfficientNet was proposed through experiments, while earlier research had focused typically on expanding one of these dimensions to enhance performance. Figure 2 represents the architecture of EfficientNet. Particularly, they formulated the problem description for exploring the relationships between the width, depth, and resolution of the network to achieve model accuracy. Consider the entire net as N, and the ith layer is formulated by $Y_{i}=F_{i}\left(X_{i}\right)$, where $F_{i}$ represents the operator, $Y_{i}$ denotes the output tensor, and $X_{i}$ indicates the input tensor. Where N has k convolution layers, $=F_{k}⊙\ldots ⊙F_{2}⊙F_{1}\left(X_{1}\right)={⊙}_{j=1\ldots ,k}F_{j}\left(X_{i}\right)$. The convolution layer is generally divided into similar architecture phases, hence N is formulated as:

(1) $$N={⊙}_{i=1\ldots s}F_{i}^{L_{i}}\left(X_{\langle H_{i},{\;W}_{i},C_{j}\rangle }\right)\;$$

In Equation (1), i refers to the stage index, $F_{i}^{L_{i}}$ denotes the convolution layer of ith stages, $F_{i}$ repeats $L_{i}$ times, and $\langle H_{i},{\;W}_{i},C_{i}\rangle$ shows the shape of the input images.

The research workers established some constraints involving fixing the fundamental architecture of the network, which imposes equivalent scaling on each layer and incorporates computation and memory constraints to decrease the search range. Consequently, the scaling of the network is enhanced by multiplying the baseline network as F^i, L^i,H^i, ^ Wi, and C^i with the constant magnification:

$$\underset{d,w,r}{\max}Accuracy\left(N\left(d,\;w,r\right)\right)$$

(2) $$s.t.N\left(d,w,r\right)={⊙}_{i=1,\ldots s}{\hat{F}}_{i}^{\hat{L_{i}}}\left(X_{\langle r\times {\hat{H}}_{i},r\times {\hat{W}}_{i},r\times {\hat{C}}_{i}\rangle }\right)\;$$

Memory (N)≤target_memory

FLOPS (N)≤target_flops

In Equation (2), w, d, and r signify the coefficients for scaling the width, depth, and resolution of the network.

The authors presented a compound scaling method after conducting an experiment that involved modifying only one dimension simultaneously, along with adjusting each of the three dimensions at a time. This technique includes a compound co-efficient ϕ to equally scale the resolution, width, and depth of the network:

$$depth:\;d=\alpha^{\phi }$$

$$width:\;w=\beta^{\phi }$$

$$depth:\;r=\gamma^{\phi }$$

(3) $$s.t.\alpha \cdot \beta^{2}\cdot \gamma^{2}\approx 2,\;where\;\alpha ,\beta ,\gamma \geq 1\;$$

In Equation (3), α, β, and γ are the constants representing a small grid search.

### 3.3. Hyperparameter Tuning Using AOA

To adjust the parameters related to the EfficientNet, the AOA is used. The concept behind the AOA technique is to perform mathematical operations such as addition, division, subtraction, and multiplication operators [30]. AOA is a basic structure with lower computation difficulty, and it can be associated with a sine-cosine algorithm (SCA). Assume that M&D companies are turning out large phases in each iteration; the exploration stage is where most of the work is performed.

(4) $$X_{i}\left(l+1\right)=\left\{\begin{array}{c} Xb\left(t\right)/(MOP+eps).\left(\left(UB-LB\right)\mu +LB\right),rand<0.5\\ Xb\left(t\right)MOP.\left(\left(UB-LB\right)\mu +LB\right),rand\geq 0.5 \end{array}\right.\;$$

where eps referring to the simple positive number and the constant coefficient represented as 1 (0.499) are two factors from the proposal model. *MOP* includes a nonlinear reduction from 0 to 1 as the iterations progress.

(5) $$MOP=1-{\left(\frac{t}{T}\right)}^{\frac{1}{\alpha }}\;$$

where α is a constant value fixed as 5. Note that both the M and D operators in Equation (5) generate a high random starting point for the best search agent. At the same time, the S and A operators are employed to devote greater attention to local exploitation, thus decreasing the count of stages beneath the search space.

The right equilibrium between use and discovery is critical to the accomplishment of maximum efficiencies in any model. The AOA parameter was utilized to switch between exploitation and exploration at each iteration.

(6) $$MOA\left(t\right)=Min+t\left(\begin{array}{c} \frac{Max-Min}{T} \end{array}\right)\;$$

In Equation (6), Min and Max indicate constant values. According to Equation (6), *MOA* enhances Min to Max. Thus, the search agent has an additional chance to conduct exploration in the searching range; then, the search agent is very likely to conduct a search near the optimal position.

The AOA technique produces an FF to enhance classification accuracy. It shows a positive integer for describing the real-time accuracy of the possible performance. The classifier error rate is supposed to be FF, and its minimization is the goal.

(7) $$fitness\left(X_{i}\right)=ClassifierErrorRate\left(X_{i}\right)\;=\frac{No.\;of\;misclassified\;instances\;}{Total\;no.\;of\;instances\;}*100\;$$

### 3.4. Skin Cancer Detection Using Optimal SDAE Model

For skin cancer classification, the SDAE model is applied. Autoencoders (AEs) are allowed to convert high-dimension input data into low-dimension feature representations [31]. For improved robustness of AE, the DAE is capable of mapping real data instances $x_{i}$ for corrupted instances ${\tilde{x}}_{i}$. Stacking multiple DAEs allows the input data that were compressed as distinct hidden spaces to be extracted for in-depth features. Therefore, the resultant layer $z_{i}^{L}$ of the SDAE is expressed as Equation (8):

(8) $$z_{i}^{L}=\phi^{L}(W^{L}\left(\cdots \phi_{1}\left(W^{1}{\tilde{x}}_{i}+b^{1}\right)\ldots )+b^{L}\right)\;$$

where ∗i denotes the corrupted input for $x_{i},\;W^{1},\;W^{2},\ldots ,W^{L}$ implies the weighted matrix, $b^{1},b^{2},\ldots ,b^{L}$ represents the bias vectors, and $\phi^{1},\phi^{2},\ldots ,\phi^{L}$ stands for the activation functions like Relu, Sigmoid, and Tanh. L signifies the count of hidden layers (HLs), with L=1 for the input layer and L=L for the resultant layer. By diminishing the error among the original input and reconstructed output, the main function of the SDAE is expressed as Equation (9):

(9) $$\zeta =\frac{1}{n}\sum_{i=1}^{n}{\|x_{i}-\phi^{L}(W^{L}(\cdots \phi^{1}(W^{1}{\tilde{x}}_{i}+b^{1})\ldots )+b^{L})\|}^{2}\;$$

Compared to the simple AE and DAE, the SDAE is a specific hierarchical model for learning the feature representation from depth in the corrupted input.

Finally, the DFA is utilized for the hyperparameter selection of the SDAE model. DFA is a recent metaheuristic technique that drew its inspiration from the static and dynamic strategies of crowding [32]. Both steps in the metaheuristic algorithm are called exploitation and exploration. DFs form small groups and fly in dissimilar regions as a static group. In the static group, DFs fly in one direction and in large groups, which are desired behaviors from the exploitation stage. To inspire the behaviors of DFs, five fundamental rules, three of which are developed by Reynold and two novel concepts, are discussed in detail:

Separation, which represents the avoidance of people’s contact with others, is given as follows.

(10) $$S_{i}=-\sum_{j=1}^{N}X-X_{i}\;$$

where the measured tap of transformer X signifies the individual location, Xj refers to the amount of power of resources in the jth location, and N shows the number of measurements.

Alignment: Compared to the total tap transformer measurement, this implies the amount of the tap transformer at different hours.

(11) $$A_{j}=\frac{\sum_{j=1}^{N}Vj}{N}\;$$

In Equation (11),  Vi denotes the number of transformer j and N refers to the amount of transformer tap measurements.

Cohesion: This implies the quantity of passing power measured in relation to the overall amount of measured powers in various hours.

(12) $$C_{j}=\frac{\sum_{j=1}^{N}Xj}{N}-X\;$$

In Equation (12), Xj denotes the amount closer to the reference value and  X shows the transformer tap rate.

Attraction: The principal objective is to maintain survival; consequently, each individual should be attracted to the food sources:

(13) $$F_{i}=X^{+}-X\;$$

In Equation (13), X denotes the reduction in transformer tap loss and $X^{+}$ is the power transmission from the network.

Distraction: This means staying away from the enemy that is shown below.

(14) $$E_{i}=X^{-}-X\;$$

In Equation (14), X denotes the location of the enemy and X shows the location of individuals.

Position vector X and the step length vector are the two vectors considered for updating the location of artificial DFs and simulating their movement.

(15) $$\Delta X_{t+10}=\left(\begin{array}{c} sS_{i}+aA_{i}+cC_{j}+\\ fF_{i}+eE_{i} \end{array}\right)+w\Delta X_{t}\;$$

In Equation (15), the Δx step length vector is the same as the speed vector in PSO, and based on the PSO technique, the DFA is developed. A denotes the alignment value based on the ith load and A indicates the co-efficient related to the direction; s denotes the number of transformer taps from the presence of scattered production; $S_{i}$ denotes the separation rate compared with the ith loss; the value f represents the nutrition factor; and $f_{i}$ indicates the food source for the ith load. The conditions of the tap transformer regarding the passing power are noted by i, w implies the inertia weight, and t shows the repetition count of the model. c shows the cohesion coefficient and $C_{i}$ denotes the cohesion value connected to i. e indicates the deviation of power transmitted, $E_{i}$. After evaluating the step vector, the position vector is evaluated using the following expression.

(16) $$\Delta X_{t+1}=X_{t}+\Delta X_{t+1}\;$$

## 4. Results Analysis

The proposed model is simulated using the Python 3.8.5 tool on a PC with the following specifications: i5-8600k, GeForce 1050 Ti 4 GB, 16 GB RAM, 250 GB SSD, and 1 TB HDD. The parameter settings are given as follows: learning rate: 0.01, dropout: 0.5, batch size: 5, epoch count: 50, and activation: ReLU. For experimental validation, 80:20 and 70:30 ratios of training/testing data are used.

In this study, the performance validation of the ODL-SCDC algorithm has been tested on the ISIC database including distinct classes, namely, Angioma (ANG) (21 images), Nevus (NEV) (46 images), Lentigo NOS (LNOS) (41 images), Solar Lentigo (SLG) (68 images), Melanoma (MEL) (51 images), Seborrheic Keratosis (SKT) (54 images), and Basal Cell Carcinoma (BCC) (37 images). Table 1 represents the details of the database.

**Figure 3:**
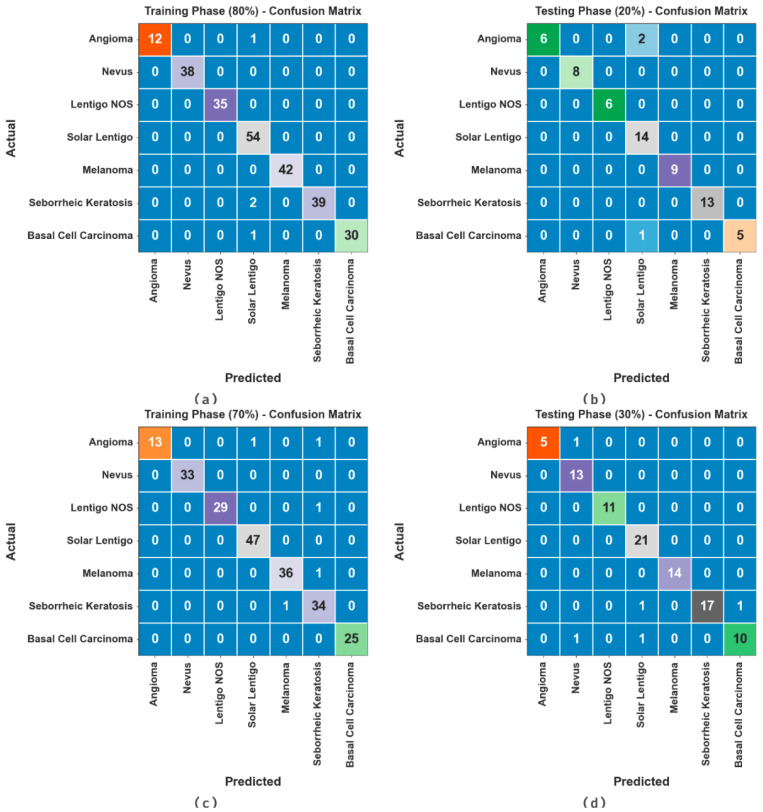
Confusion matrices at (**a**,**b**) 80:20 of TR phase/TS phase and (**c**,**d**) 70:30 of TR phase/TS phase.

Figure 3 exhibits the confusion matrices attained by the ODL-SCDC methodology at 80:20 and 70:30 of the TR phase/TS phase. The outcome inferred the effective recognition and classification of all seven classes.

**Figure 4:**
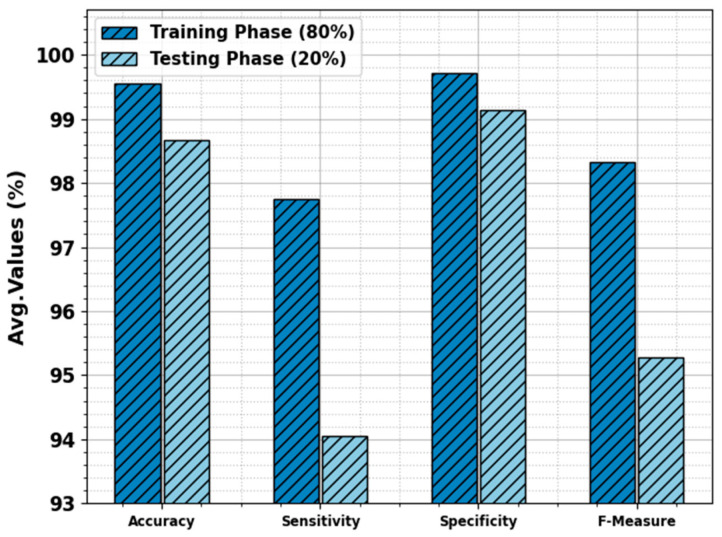
Average of ODL-SCDC algorithm at 80:20 of TR phase/TS phase.

The skin cancer classification result of the ODL-SCDC technique is provided at 80:20 of the TR phase/TS phase in Table 2 and Figure 4. The experimental values inferred that the ODL-SCDC technique gains enhanced performance under all classes. With 80% of the TR phase, the ODL-SCDC technique offers average $accu_{y}$, $sens_{y}$, $spec_{y}$, and $F_{measure}$ of 96.55%, 97.74%, 99.71%, and 98.33%, respectively. Additionally, with 20% of the TS phase, the ODL-SCDC system gains average $accu_{y}$, $sens_{y}$, $spec_{y}$, and $F_{measure}$ of 98.66%, 94.05%, 99.14%, and 95.28%, correspondingly.

**Figure 5:**
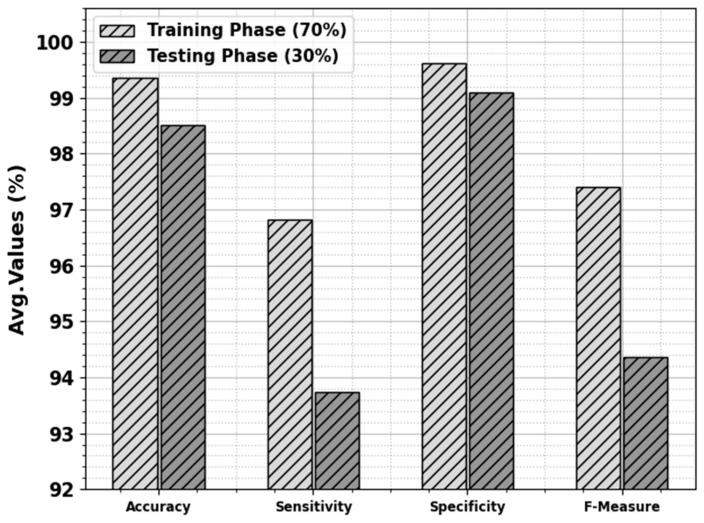
Average of ODL-SCDC algorithm at 70:30 of TR phase/TS phase.

The skin cancer classification outcome of the ODL-SCDC technique is provided at 70:30 of the TR phase/TS phase in Table 3 and Figure 5. The simulation values implied that the ODL-SCDC method obtains higher outcomes under all classes. With 70% of the TR phase, the ODL-SCDC system attains average $accu_{y}$, $sens_{y}$, $spec_{y}$, and $F_{measure}$ of 99.36%, 96.82%, 99.61%, and 97.41%, correspondingly. Furthermore, with 30% of the TS phase, the ODL-SCDC algorithm gains average $accu_{y}$, $sens_{y}$, $spec_{y}$, and $F_{measure}$ of 98.51%, 93.73%, 99.10%, and 94.37%, correspondingly.

**Figure 6:**
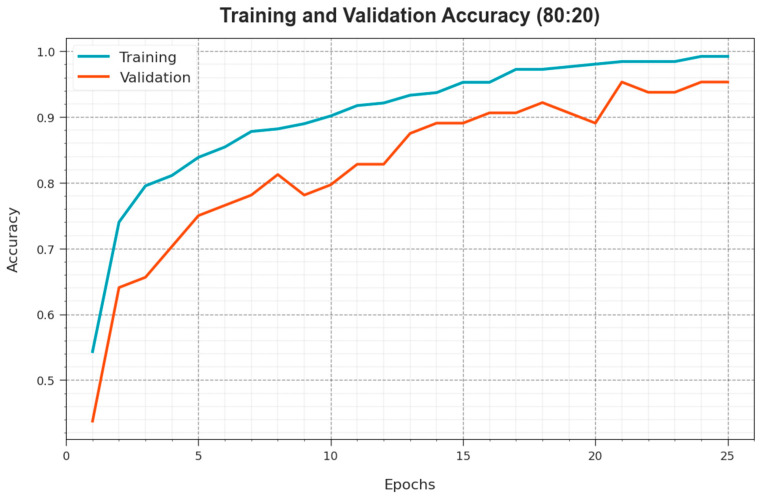
$Accu_{y}$ curve of ODL-SCDC approach at 80:20 of TR phase/TS phase.

To calculate the performance of the ODL-SCDC approach at 80:20 of the TR phase/TS phase, TR and TS $accu_{y}$ curves are determined, as revealed in Figure 6. The TR and TS $accu_{y}$ curves establish the performance of the ODL-SCDC model over several epochs. The figure provides meaningful details regarding the learning task and generalisation abilities of the ODL-SCDC model. With an enhanced epoch count, it is noticed that the TR and TS $accu_{y}$ curves are improved. It is experimental that the ODL-SCDC algorithm obtains better testing accuracy which has the capability of recognizing the patterns in the TR and TS data.

**Figure 7:**
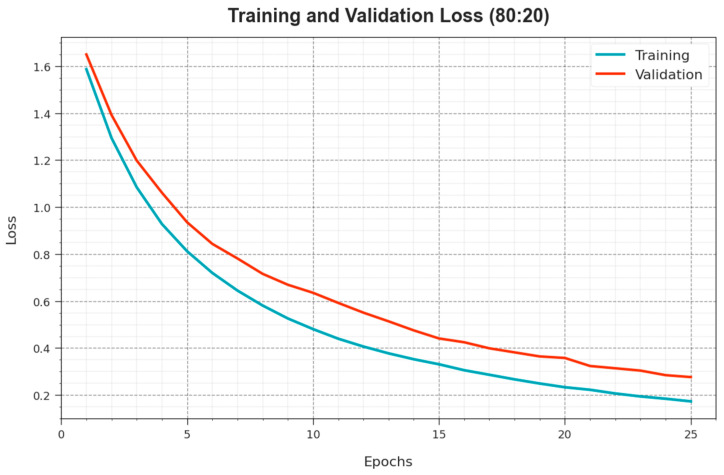
Loss curve of ODL-SCDC approach at 80:20 of TR phase/TS phase.

Figure 7 exhibits the overall TR and TS loss values of the ODL-SCDC algorithm at 80:20 of the TR phase/TS phase over epochs. The TR loss exhibits that the method loss is minimal over epochs. Primarily, the loss values are lesser as the model modifies the weight to minimize the prediction error on the TR and TS data. The loss curves demonstrate the extent to which the model fits the training data. It is detected that the TR and TS loss is steadily decreased and depicted that the ODL-SCDC system effectually learns the patterns exhibited in the TR and TS data. It is also observed that the ODL-SCDC methodology modifies the parameters to decrease the discrepancy between the prediction and the original training label.

**Figure 8:**
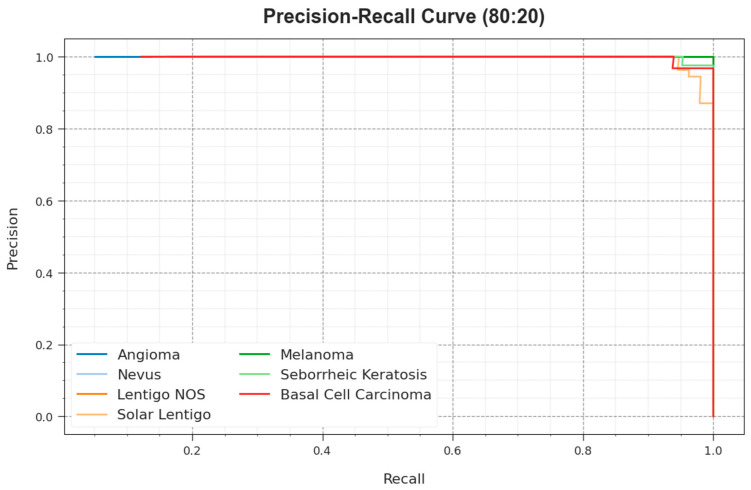
PR curve of ODL-SCDC system at 80:20 of TR phase/TS phase.

The precision–recall curve of the ODL-SCDC system at 80:20 of the TR phase/TS phase is demonstrated by plotting precision against recall as defined in Figure 8. The outcome confirms that the ODL-SCDC approach reaches higher precision–recall outcomes under all classes. The figure represents that the model learns to recognize various classes. The ODL-SCDC model accomplishes improved results in the recognition of positive instances with minimal false positives.

**Figure 9:**
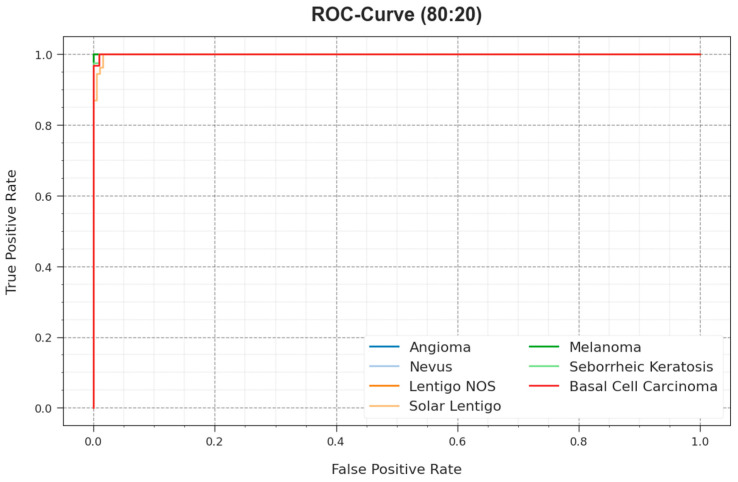
ROC curve of ODL-SCDC approach at 80:20 of TR phase/TS phase.

The ROC curves offered by the ODL-SCDC model at 80:20 of the TR phase/TS phase are illustrated in Figure 9, which have the ability the discriminate the class labels. The figure implies valuable insights into the trade-off between the TPR and FPR rates over distinct classification thresholds and varying numbers of epochs. It presents the accurate predictive performance of the ODL-SCDC system on the classification of various classes.

**Figure 10:**
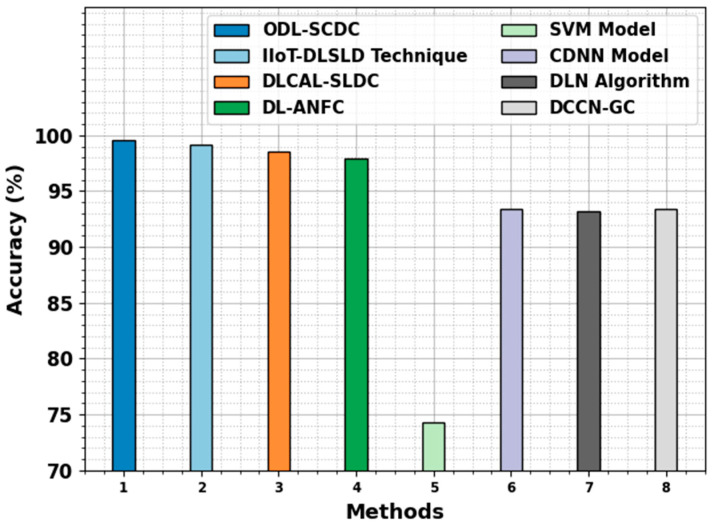
$Accu_{y}$ outcome of ODL-SCDC algorithm with other approaches.

In Table 4, a comprehensive comparison study of the ODL-SCDC technique is made [1]. Figure 10 represents the comparative results of the ODL-SCDC technique in terms of $accu_{y}$. Based on $accu_{y}$, the ODL-SCDC technique gains an increasing $accu_{y}$ of 99.55%, whereas the IIOT-DLSLD, DLCAL-SLDC, DL-ANFC, SVM, CDNN, DLN, and DCCN-GC models obtain decreasing $accu_{y}$ values of 99.20%, 98.50%, 97.90%, 74.30%, 93.40%, 93.20%, and 93.40%, respectively.

**Figure 11:**
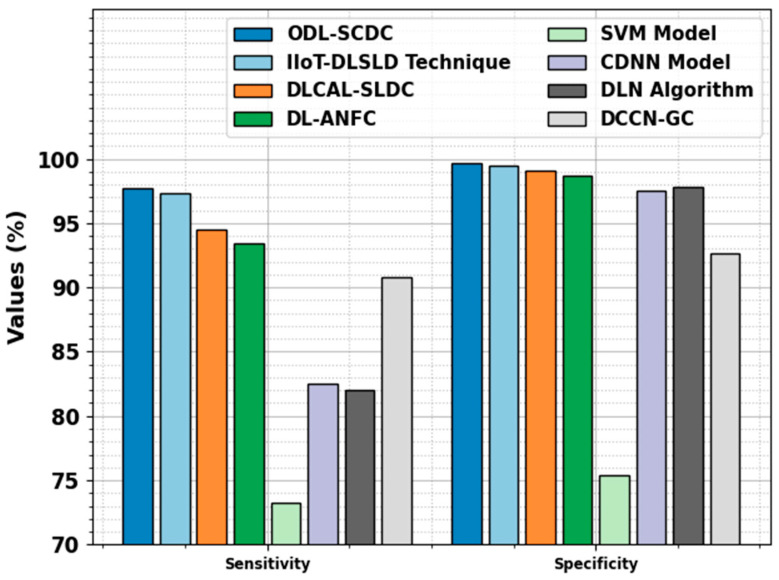
$Sens_{y}$ and $spec_{y}$ outcomes of ODL-SCDC algorithm with other approaches.

Figure 11 signifies the comparative outcomes of the ODL-SCDC approach in terms of $sens_{y}$ and $spec_{y}$. Based on $sens_{y}$, the ODL-SCDC technique gains a higher $sens_{y}$ of 97.74%, whereas the IIOT-DLSLD, DLCAL-SLDC, DL-ANFC, SVM, CDNN, DLN, and DCCN-GC systems obtain decreasing $sens_{y}$ values of 97.30%, 94.50%, 93.40%, 73.20%, 82.50%, 82%, and 90.80%, correspondingly. Based on $spec_{y}$, the ODL-SCDC methodology achieves a higher $spec_{y}$ of 99.71%, whereas the IIOT-DLSLD, DLCAL-SLDC, DL-ANFC, SVM, CDNN, DLN, and DCCN-GC algorithms obtain lesser $spec_{y}$ values of 99.50%, 99.10%, 98.70%, 75.40%, 97.50%, 97.80%, and 92.70%, correspondingly.

**Figure 12:**
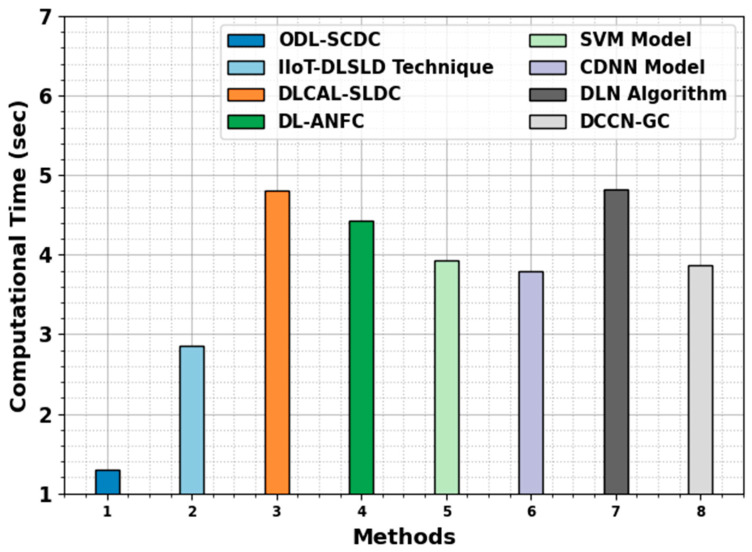
CT outcome of ODL-SCDC algorithm with other approaches.

Lastly, the computation time (CT) results of the ODL-SCDC technique are compared with recent models in Table 5 and Figure 12. The experimental outcomes infer the lowest CT value of the ODL-SCDC technique with 1.30 s. On the other hand, the IIoT-DLSLD, DLCAL-SLDC, DL-ANFC, SVM, CDNN, DLN, and DCCN-GC models obtain increasing CT values. Therefore, the ODL-SCDC technique exhibits effectual performance of skin cancer classification.

## 5. Conclusions

In this article, we have designed and developed an automated skin cancer classification and detection model using the ODL-SCDC technique in the IoT environment. The goal of the ODL-SCDC technique is to exploit metaheuristic-based hyperparameter selection approaches with a DL model for skin cancer classification. To achieve this, the ODL-SCDC technique performs a series of processes such as WF-based processing, EfficientNet-based feature extraction, AOA-based hyperparameter tuning, SDAE-based classification, and DFA-based parameter tuning. In addition, the ODL-SCDC system involves the AOA with the EfficientNet algorithm for feature extraction. For skin cancer detection, the SDAE classification model has been used. Lastly, the DFA is utilized for the optimal hyperparameter selection of the SDAE algorithm. The simulation validation of the ODL-SCDC algorithm has been tested on a benchmark skin lesion database. The extensive results reported the enhanced performance of the ODL-SCDC technique with other approaches with respect to distinct measures.

## Figures and Tables

**Table 1:** Database details.

Class	No. of Images
Angioma	21
Nevus	46
Lentigo NOS	41
Solar Lentigo	68
Melanoma	51
Seborrheic Keratosis	54
Basal Cell Carcinoma	37
Total Number of Images	318

**Table 2:** Skin cancer classifier outcome of ODL-SCDC algorithm at 80:20 of TR phase/TS phase.

Class	$Accu_{y}$	$Sens_{y}$	$Spec_{y}$	$F_{Measure}$
TR Phase (80%)				
Angioma	99.61	92.31	100.00	96.00
Nevus	100.00	100.00	100.00	100.00
Lentigo NOS	100.00	100.00	100.00	100.00
Solar Lentigo	98.43	100.00	98.00	96.43
Melanoma	100.00	100.00	100.00	100.00
Seborrheic Keratosis	99.21	95.12	100.00	97.50
Basal Cell Carcinoma	99.61	96.77	100.00	98.36
Average	99.55	97.74	99.71	98.33
TS Phase (20%)				
Angioma	96.88	75.00	100.00	85.71
Nevus	100.00	100.00	100.00	100.00
Lentigo NOS	100.00	100.00	100.00	100.00
Solar Lentigo	95.31	100.00	94.00	90.32
Melanoma	100.00	100.00	100.00	100.00
Seborrheic Keratosis	100.00	100.00	100.00	100.00
Basal Cell Carcinoma	98.44	83.33	100.00	90.91
Average	98.66	94.05	99.14	95.28

**Table 3:** Skin cancer classifier outcome of ODL-SCDC algorithm at 70:30 of TR phase/TS phase.

Class	$Accu_{y}$	$Sens_{y}$	$Spec_{y}$	$F_{Measure}$
TR Phase (70%)				
Angioma	99.10	86.67	100.00	92.86
Nevus	100.00	100.00	100.00	100.00
Lentigo NOS	99.55	96.67	100.00	98.31
Solar Lentigo	99.55	100.00	99.43	98.95
Melanoma	99.10	97.30	99.46	97.30
Seborrheic Keratosis	98.20	97.14	98.40	94.44
Basal Cell Carcinoma	100.00	100.00	100.00	100.00
Average	99.36	96.82	99.61	97.41
TS Phase (30%)				
Angioma	98.96	83.33	100.00	90.91
Nevus	97.92	100.00	97.59	92.86
Lentigo NOS	100.00	100.00	100.00	100.00
Solar Lentigo	97.92	100.00	97.33	95.45
Melanoma	100.00	100.00	100.00	100.00
Seborrheic Keratosis	97.92	89.47	100.00	94.44
Basal Cell Carcinoma	96.88	83.33	98.81	86.96
Average	98.51	93.73	99.10	94.37

**Table 4:** Comparative outcome of ODL-SCDC algorithm with other approaches.

Methods	$Sens_{y}$	$Spec_{y}$	$Accu_{y}$
ODL-SCDC	97.74	99.71	99.55
IIoT-DLSLD Technique	97.30	99.50	99.20
DLCAL-SLDC	94.50	99.10	98.50
DL-ANFC	93.40	98.70	97.90
SVM Model	73.20	75.40	74.30
CDNN Model	82.50	97.50	93.40
DLN Algorithm	82.00	97.80	93.20
DCCN-GC	90.80	92.70	93.40

**Table 5:** CT outcome of ODL-SCDC algorithm with other approaches.

Methods	Computational Time (s)
ODL-SCDC	1.30
IIoT-DLSLD Technique	2.85
DLCAL-SLDC	4.80
DL-ANFC	4.43
SVM Model	3.93
CDNN Model	3.80
DLN Algorithm	4.82
DCCN-GC	3.87

## Data Availability

Data sharing does not apply to this article as no datasets were generated during the current study.
